# Awareness Related to Cardiometabolic Diseases: A Cross-Sectional Study in Southern Vietnam

**DOI:** 10.3390/ijerph181910209

**Published:** 2021-09-28

**Authors:** Chau Minh Nguyen, Cornelia Melinda Adi Santoso, Duyen Thi Huong Vu, Gergő Szőllősi, Róbert Bata, Judit Zsuga, Attila Csaba Nagy

**Affiliations:** 1 Faculty of Public Health, University of Debrecen, 4032 Debrecen, Hungary; nguyen.chauminh96@gmail.com (C.M.N.); cornelia.melinda@sph.unideb.hu (C.M.A.S.); szollosi.gergo@sph.unideb.hu (G.S.); bata.robert@sph.unideb.hu (R.B.); zsuga.judit@med.unideb.hu (J.Z.); 2 Training and Direction of Healthcare Activities Center, Cho Ray Hospital, Ho Chi Minh City 72713, Vietnam; huongduyen1966@gmail.com

**Keywords:** awareness, hypertension, type 2 diabetes, prevention

## Abstract

Background: the prevalence of cardiometabolic diseases (CMDs), such as type 2 diabetes mellitus (T2DM) and hypertension, is increasing rapidly in developing countries. This study aims to assess the awareness of CMD among a selected population in Vietnam. Method: a cross-sectional random sample of 402 Vietnamese citizens in two districts (Thu Duc and 12th district) in Ho Chi Minh City were interviewed. Data on knowledge, attitude, and preventive behavior (KAB) of the two conditions were collected through an interview-based questionnaire. Results: the mean (± SD) age was 47.75 (± 15.61) years, and around 60.2% were female. Multiple logistic regression was performed to explore the association of sociodemographic factors, disease status, and awareness of the CMD. Females showed better awareness than males (OR = 3.89 (1.28–11.78)), and those with T2DM and hypertension had a significantly better awareness (OR = 8.33 (2.44–28.37)) than those without CMD. Conclusion: the awareness of CMD in our sample was poor. An extensive effort to increase awareness of CMD prevention is needed. Future studies and interventions can be developed more efficiently by targeting the right population.

## 1. Introduction

Cardiometabolic disease (CMD) is described as the combination of many disorders, of which cardiovascular diseases (CVDs) and diabetes mellitus (DM) are two of the most crucial components. In 2019, CVDs were responsible for 17.9 million deaths, estimated to be the number one cause of death globally [1]. DM was ranked as the eighth leading cause of death in 2019, responsible for 4.2 million deaths [2,3]. According to the World Health Organization (WHO) estimation in 2020, there were 1.13 billion adults over the world who live with hypertension. However, less than one out of five hypertensive patients are under proper control, leading hypertension to become one of the major causes of premature death worldwide [4]. The majority of CMD risk factors are highly modifiable, especially hypertension and type 2 DM (T2DM) [5,6]. In the past, mostly older people were affected. However, due to rapid urbanization, sedentary lifestyles, and unhealthy diets, these diseases have been frequently diagnosed in younger patients in the past few years. Specifically, in Vietnam (2012), the prevalence of hypertension was 25.1% (28.3% vs. 23.1% in males and females, respectively), and the prevalence of diabetes was 6.0% [7,8,9]. Lifestyle modification, together with regular checkups and medication support (if necessary) is essential to prevent and control these conditions [10,11].

Ho Chi Minh City (HCMC) is the second largest and most populous city in Vietnam, with a population of approximately 9 million people in 2019. The rapid socioeconomic growth of the city is accompanied by lifestyle changes that may influence the burden of CMD. According to the 2014 national health survey, HCMC has the highest increasing rate of diabetes in the country [12]. While the healthcare system in HCMC has improved in the past decade, it still primarily focuses on enhancing medical treatments. There are only a few studies conducting health promotion programs on the primary prevention of CMD in Vietnam [13,14]. Furthermore, few of the studies have measured the “knowledge, attitude, and behavior” (KAB) of CMD [15]. Understanding people’s KAB is essential because KAB influences the individual’s ability to make decisions about their own healthcare. Lack of health literacy may cause people to forgo preventive healthcare or to engage in poor health behaviors [16,17].

This study aimed to analyze the awareness of cardiometabolic diseases (i.e., hypertension and type 2 diabetes mellitus) in the selected population in HCMC.

## 2. Materials and Methods

### 2.1. Study Design and Setting

This cross-sectional, descriptive study used an 84 question–interviewer-filled questionnaire in multiple communes, of two conveniently chosen districts (district 12 and Thu Duc) in HCMC, Vietnam.

### 2.2. Sampling and Sample Size

The sample size was calculated using the “sampsi” command in STATA, with the following reference mean (± SD) systolic blood pressures from a previous study: males 126.0 (± 18.3) and females 120.4 (± 18.5), respectively [18]. It gave the result of 227 participants per district since the study required 454 participants. To avoid rejection, inappropriate answers, and ineligible households, 10% additional participants were added; hence, the study required 500 participants. After the data collection, only 402 participants were available to participate (response rate: 80.4%).

Participants were randomly selected from households in the designated areas. We employed a two-stage sampling method. The first stage was to conveniently select the streets in the two districts. The second stage was to use systematic random sampling to choose the households. The starting point of selecting the households was the first house on the right side of the streets. From this point, we chose every second household that was on the right side of the previously selected household. The randomly selected households had to have one or more eligible members (18 years old or older). All participants were informed of the study’s purpose and procedure before signing the informed consent form to participate. Upon signing the informed consent form, the participants agreed to share their personal medical record books and to precisely answer the questions, which were asked by the interviewer. Participation in the study was voluntary, and no identifying information was elicited. To ensure anonymity and confidentiality, each participant was assigned a code number, and all data were stored in locked files, accessible only to the researchers, until the completion of the study. The implementation of this study’s data collection was approved by the University of Public health, Vietnam (444/DHYTCC).

### 2.3. Questionnaire

The questionnaire was based on the 2011 questionnaire on T2DM patients’ knowledge of the Vietnamese Ministry of Health [19].

The first eight questions requested the participants’ sociodemographic data and the disease status, which was recorded based on their personal medical histories.

The next 42 questions assessed the participants’ knowledge of CMD, 12 questions assessed their attitudes, and 22 questions were related to their behaviors. To acquire precise information, three public health workers were chosen to be the interviewers, and they were trained specifically to perform the study questionnaire.

Within the 42 questions of general knowledge, 23 questions concerned the knowledge of T2DM, and the other 19 questions concerned the knowledge of hypertension. Knowledge of both conditions was required, to be categorized as having good knowledge of cardiometabolic diseases.

The next 12 questions assessed the participants’ attitudes regarding the dangerousness of their conditions and their adherence to medical advice.

The behavior questions were asked at the beginning of the interview to prevent the participants from answering them based on the knowledge questions. The 22 questions in this part assessed the participants’ lifestyles and habits. Healthy diet guidelines and physical activity (PA) recommendations (at least 30 min of moderate or intense PA per day) were determined based on the World Health Organization (WHO) and the Centers for Disease Control and Prevention (CDC) guidelines [20,21]. The use of cigarette and alcohol were also assessed.

### 2.4. Data Analysis

The general awareness of CMD was evaluated on the combination of three factors: general knowledge of the diseases and their prevention (its risk factors and the conditions), the attitude of the participants, and their behaviors (such as diet, physical activity, medical seeking, etc.). A participant received one point for each category if the score was >70%, which was in accordance with previous studies [15,22,23,24,25]. Scoring in at least two out of three categories was considered as having a good degree of awareness concerning cardiometabolic diseases.

The data analysis was performed by using STATA (version 13.0, Stata Corp, College Station, TX, USA) and Microsoft Excel 2016. For statistical purposes, all collected data were set into binary variables. The significance threshold was set at <0.05. Multiple logistic regression was used to calculate the odds ratios (ORs) and the corresponding 95% confidence interval (95%CI) to determine the association of sociodemographic characteristics with KAB and the general awareness of the participants.

## 3. Results

The demographic characteristics of the sample are shown in Table 1. The mean (± SD) age was 47.75 (± 15.61) years. Around 60.2% were female and 30.8% had only primary education or below. The mean (± SD) of body mass index, systolic blood pressure, diastolic blood pressure, and blood glucose were 22.94 (± 3.19) kg/m2, 122.18 (± 15.31) mmHg, 84.3 (± 11.60) mmHg, and 6.43 (± 4.61) mmol/L, respectively. According to the past medical records, 17.7% of them had hypertension only, 2.5% had T2DM only, and 6.7% were diagnosed with both conditions.

The percentages of participants with good vs. bad KAB and awareness is shown in Table 1. Around 11.0% of the participants had relatively good knowledge of CMD; 11.9% and 35.1% of the participants had good knowledge of T2DM and hypertension, respectively. About 26.1% of the participants showed a good attitude regarding CMD, and 5% had good preventive behavior. After combining knowledge, attitude, and behavior, only 8% of the participants had good awareness of CMD.

The descriptive statistics of KAB and awareness of the study participants are shown in Table 2. Only 44 participants (10.95%) had good general knowledge of both T2DM and hypertension, including definitions, risk factors, preventions, and complications. Separately, 48 participants (11.94%) had good knowledge of T2DM and 141 participants (35.07%) had good knowledge of hypertension. No more than 105 participants (26.12%) had a good attitude. Only 20 participants (4.98%) had good lifestyles of healthy diets and physical activity for at least 30 min a day, over four days per week. By combining the three main factors (KAB), with a score of two out of three factors, 32 participants (7.96%) had good awareness of CMD awareness.

The associations between the demographic characteristics of the study population and their knowledge, attitude, behavior (KAB), and their general awareness (GA) is shown in Table 3. Older participants showed significantly better attitude (OR (95%CI) = 1.04 (1.02–1.06)) and behavior (1.07 (1.02–1.12)). Females tended to have better knowledge (2.80 (1.23–6.33)) and awareness compared to males (3.89 (1.28–11.78)), but separately, no significant association was found between gender and attitude or behavior. Those who finished tertiary or higher education had significantly better knowledge (3.35 (1.25–9.00)) than those who finished only primary education or less. However, the less educated group (primary or below) showed a significantly better attitude compared to the secondary and higher educated group (0.46 (0.26–0.79) and 0.41 (0.20–0.86), respectively). No association was found between employment, marital status, and KAB or GA. Those diagnosed with T2DM showed much better knowledge (10.61 (2.49–45.10)) than those without a CMD. Those with hypertension tended to have better attitudes (2.06 (1.10–3.89)) than those without CMD. Lastly, those diagnosed with both T2DM and hypertension had significantly better knowledge (7.70 (2.53–23.42)) and awareness (8.33 (2.44–28.37)), compared to those without any CMD.

## 4. Discussion

General awareness of CMD was found poor among the study population. The two notable factors associated with CMD awareness were gender and CMD status. Further examining the factors associated with the specific domains of awareness, age was found to be positively associated with attitude and behavior, female gender was associated with better knowledge, and having both CMD conditions was associated with better knowledge and awareness. Interestingly, we found that educational level was positively associated with knowledge, but negatively associated with attitude.

Our finding of poor awareness of CMD was similar to previous studies, which showed low awareness of T2DM in a region of Northern Vietnam, and low awareness of hypertension at a national level [7,26]. The stereotype in Vietnam is that treatment is the definite answer to diseases, and prevention is only recommended and optional. Hence, even though they agreed that the diseases could pose a serious threat to their health, or even their lives, they were not concerned since they were not yet ill. Despite the existence of many health promotion programs aimed to improve the public knowledge and awareness of CMD in Vietnam [7,27,28,29], our findings suggest that the programs might not be effective nor able to change the public attitudes. These programs might either target the wrong population or not cover a large enough population.

In line with previous studies [7], women were more likely to have better knowledge and overall awareness of CMD compared with men. With the stereotype that women always cared for their health better than men, they were more likely to seek information on diseases and practice healthy lifestyles [30]. While age was not associated with the knowledge and general awareness of CMD, we found that the older participants had a better attitude and behavior than the younger participants. Elders may care more for their health, and thus have better adherence to medical advice and better lifestyle than younger participants. Furthermore, the fact that people only cared about their health when they had the diseases (having positive CMD status) was associated with better knowledge and awareness.

Our study found that higher educational level was associated with better knowledge, but worse attitude, and was found as an indicator for higher socioeconomic status (SES). People with better SES in Vietnam may be more likely to be exposed to a western lifestyle, such as fast-food or sugar-rich diets, as they are more able to afford them [31,32]. They are also more likely to have beliefs that they can afford the medical treatments and, thus, they may not have good attitude towards prevention. On the other hand, less educated people may be more afraid of the conditions, possibly because they know that these conditions are difficult and costly to treat. Hence, they would be more receptive to the doctor’s instructions and the information about health promotion programs.

Our study has some limitations. First, the sampling method used in this study was ‘convenience sampling’, and we did not reach the pre-specified sample size. Thus, this study might affect our result and the finding could only be internally valid and not representative of the general population in HCMC. Second, the questionnaire was self-reported, which may lead to social desirability bias. Despite these limitations, this study was among the few that investigated the KAB in Vietnam. The findings of this study can inspire others to carry out future research on a larger scale.

## 5. Conclusions

Our study found that general awareness of the seriousness of CMD was poor. Future public health programs should target the improvement of public awareness related to these diseases, and promotion of healthy lifestyles, especially among the young and the male population. Promoting lifestyle modifications may also be complemented by improving healthcare professionals’ skills in advising people at risk of these diseases. Achieving this mission will overall require certain changes in public health policies and a firm commitment from all governmental sectors in Vietnam.

## Figures and Tables

**Table 1:** Characteristics of participants (*n* = 402).

Variables	Population (*n* ^a^ = 402)	Only T2DM (*n* ^a^ = 10)	Only Hypertension (*n* ^a^ = 71)	Both (*n* ^a^ = 27)	No CMD (*n* ^a^ = 294)
**Age** **(Mean ± SD, years)**	47.75 (± 15.61)	53.4 (± 10.70)	58.38 (± 13.58)	66.48 (± 10.89)	43.28 (± 13.95)
**Body mass index** **(Mean ± SD, kg/m^2^)**	22.94 (± 3.19)	23.31 (± 2.46)	23.56 (± 3.78)	24.86 (± 3.45)	22.62 (± 2.95)
**Systolic blood pressure** **(Mean ± SD, mmHg)**	122.18 (± 15.31)	116.7 (± 16.44)	132.58 (± 18.18)	134.44 (± 15.82)	118.72 (± 12.58)
**Diastolic blood pressure** **(Mean ± SD, mmHg)**	84.3 (± 11.60)	75.8 (± 8.50)	83.97 (± 14.60)	83.15 (± 9.42)	79.48 (± 10.85)
**Blood glucose** **(Mean ± SD, mmol/L)**	6.43 (± 4.61)	7.09 (± 2.76)	6.73 (± 2.03)	8.27 (± 2.77)	6.16 (± 5.17)
**Gender (%)**					
Male	39.8	3.8	11.9	6.3	78.1
Female	60.2	1.7	21.5	7.0	69.8
**Education (%)**					
Primary or below	30.8	3.2	29.8	8.9	58.1
Secondary	43.8	1.1	16.5	5.7	76.7
Tertiary or higher	25.4	3.9	4.9	5.9	85.3
**Employment (%)**					
Employed	51.0	2.0	8.3	2.9	86.8
Unemployed	49.0	3.0	27.4	10.7	58.9
**Marital status (%)**					
Single	22.1	2.2	11.2	9.0	77.5
Married	77.9	2.6	19.5	6.1	71.9
**Population (%)**	100	2.5	17.7	6.7	73.1

SD: standard deviation, ^a^ *n* = the number of participants.

**Table 2:** KAB and awareness of participants on cardiometabolic diseases (*n* = 402).

Variables	Good		Bad	
	*n* ^a^	% ^b^	*n* ^a^	% ^b^
**Knowledge**				
**T2DM**	48	11.94	354	88.06
**Hypertension**	141	35.07	261	64.93
**Cardiometabolic**	44	10.95	358	89.05
**Attitude**	105	26.12	297	73.88
**Behavior**	20	4.98	382	95.02
**Awareness**	32	7.96	370	92.04

^a^*n* = the number of participants, ^b^ % = the percentage.

**Table 3:** Associations of sociodemographic characteristics with knowledge, attitude, behavior, and awareness of cardiometabolic diseases.

KAB and Awareness	Knowledge (OR (95%CI))	Attitude (OR (95%CI))	Behavior (OR (95%CI))	Awareness (OR (95%CI))
**Age**				
	1.01 (0.98–1.04)	**1.04 (1.02–1.06)**	**1.07 (1.02–1.12)**	1.03 (1.00–1.07)
**Gender**				
Female/Male	**2.80 (1.23–6.33)**	1.42 (0.81–2.50)	3.67 (0.91–14.89)	**3.89 (1.28–11.78)**
**Education**				
Secondary/Primary or below	1.67 (0.69–4.04)	**0.46 (0.26–0.79)**	0.77 (0.25–2.44)	2.01 (0.77–5.23)
Tertiary or higher/Primary or below	**3.35 (1.25–9.00)**	**0.41 (0.20–0.86)**	2.07 (0.51–8.48)	2.24 (0.65–7.76)
**Employment**				
Employed/Unemployed	0.90 (0.41–2.00)	0.70 (0.39–1.25)	0.63 (0.14–2.72)	0.59 (0.20–1.72)
**Marital status**				
Married/Single	0.60 [(0.28–1.30)	0.87 (0.46–1.64)	0.97 (0.30–3.15)	0.65 (0.26–1.65)
**CMD status**				
T2DM/No CMD	**10.61 (2.49–45.10)**	0.68 (0.13–3.58)	4.24 (0.43–42.00)	3.49 (0.37–32.67)
HTN/No CMD	1.63 (0.59–4.52)	**2.06 (1.10–3.89)**	2.86 (0.84–9.81)	2.81 (0.96–8.23)
Both/No CMD	**7.70 (2.53–23.42)**	1.54 (0.60–3.92)	1.40 (0.27–7.26)	**8.33 (2.44–28.37)**

OR: odds ratio, 95%CI: 95% confidence interval. Bold values represent significant association.

## Data Availability

The data are available upon request.
